# Warning people about the risk of AI error mitigates human acquisition of AI bias

**DOI:** 10.1186/s41235-026-00726-w

**Published:** 2026-04-30

**Authors:** Lucía Vicente, Helena Matute

**Affiliations:** 1 Department of Education Sciences, University of La Rioja, C. Luis de Ulloa, 2, 26004 Logroño, La Rioja Spain; 2 Department of Psychology, University of Deusto, Bilbao, Spain

**Keywords:** Artificial intelligence (AI), Bias, Human-AI interaction, Decision-making

## Abstract

Empirical evidence has demonstrated the power of AI to influence human decisions and the risk of humans acquiring AI biases. Therefore, there is a clear need to develop strategies to mitigate such threat. In three experiments, set in a medical context, we tested whether warning individuals about AI biases and errors could mitigate the negative impact of AI biases on their decisions and reduce the transmission of AI biases to humans. In Experiment 1, participants received explicit information about the percentage of erroneous AI recommendations but with two different framings: in terms of AI accuracy or AI risk of error. Our results showed that emphasising the risk of AI errors, more than its accuracy, reduced people’s tendency to follow incorrect AI suggestions and to acquire biases from AI. In Experiment 2, a more general warning message alerting of possible AI errors and biases was also effective in reducing bias acquisition. Experiment 3 showed that, although the warning message provided some protection against bias, participants who received AI support still made more errors than participants who completed the classification task without any assistance. Experiments 2 and 3 also investigated whether the type of error made by the AI, a false positive or a false negative, influenced participants’ tendency to adhere to its suggestions, and the effect of the warning message. However, no significant effects were found. Overall, our results highlight the importance of informing users about the risk of AI error rather than focusing solely on accuracy.

## Significance statement

Current legislation heavily relies on human oversight to mitigate the risks associated with biased or unethical AI-driven decisions, particularly in high-stakes areas, such as healthcare. However, human control over intelligent systems is not always a zero-risk solution. Research shows that individuals often over-rely in automation and may accept AI recommendations even when they are incorrect or biased. In the case of flawed AI, this reliance can lead people to reinforce existing biases or even develop new ones. Recent evidence indicates that individuals can acquire biases through repeated interactions with biased AI systems.

Our study evaluates specific interventions designed to prevent bias transmission from AI to humans. Our findings contribute to the limited understanding of strategies and conditions that promote effective collaboration between humans and AI to maximise the benefits of this technology while minimising the risk. We demonstrated that simple interventions, such as reminding users of the fallibility of AI, can enable them to make better decisions about when to use the system and when to trust their own judgement. This approach reduced the likelihood of people accepting biased recommendations from AI, and thus mitigated the risk of adopting those biases. Based on our findings, we provide recommendations for policymakers and developers on how to responsibly communicate information about AI to help users identify potential errors and biases. Our conclusions can be applied to improve AI transparency and human-AI collaboration in real-world applications.

## Warning people about the risk of AI error mitigates human acquisition of AI bias

According to regulations, human oversight will be critical in protecting people from unethical or biased decisions made by artificial intelligence (AI). For instance, the Artificial Intelligence Act (Council of the European Union, 2024) mandates human supervision of AI in high-impact areas to minimise risks to people’s health, safety, and fundamental rights. Similarly, the General Data Protection Regulation guards the human right not to be subjected to decisions based solely on automated data processing (European Parliament and the Council, 2016), particularly when such decisions can significantly affect people’s lives, such as their employment opportunities or their access to healthcare. Thus, humans stand as a front line of defence against problematic AI decisions. However, current attempts at legislation do not define the specific criteria that adequate human supervision should satisfy, how meaningful human intervention in AI decisions will be ensured, or what competencies overseers will need (Laux, 2023; Lazcoz & de Hert, 2023).

In an effective human—AI collaboration, individuals integrate accurate and helpful information from AI into their decision-making process while, at the same time, they are able to discard any incorrect or biased recommendations from the machine (De-Arteaga et al., 2020; Reverberi et al., 2022; Solans et al., 2022). As a result, such teams can potentially achieve better outcomes than both humans and AI could have achieved working independently (Inga et al., 2023; Ng et al., 2023; Patel et al., 2019; Rothfuß et al., 2023; Tschandl et al., 2020). This type of collaboration would allow people to reap the benefits of artificial intelligence models while ensuring human oversight in areas where AI accuracy may be compromised and where there is a risk of bias (Patel et al., 2019).

Against the evidence of effective collaboration, a significant body of research refutes the idea that a synergistic cooperation between humans and AI could be a common phenomenon. Humans tend to over-rely on the advice provided by artificial intelligence (Agudo & Matute, 2021; Bogert et al., 2022; Logg et al., 2019). Consequently, this can lead to people uncritically following machine advice, even if these recommendations are incorrect or biased (Goddard et al., 2011, 2012, 2014; Lyell et al., 2017; Suresh et al., 2020; Vicente & Matute, 2023). Several studies have found that incorrect AI suggestions lead people to make mistakes, even when they could have made a better decision on their own (Agudo et al., 2024; Howard et al., 2020; Vicente & Matute, 2023), a phenomenon that has also been demonstrated in high-risk contexts such as healthcare (Adam et al., 2022; Dratsch et al., 2023; Gaube et al., 2021; Jacobs et al., 2021; Rezazade Mehrizi et al., 2023). This evidence suggests that humans will not always be able to challenge poor AI decisions, which is a major obstacle to achieving the effective human oversight of AI that policy proposals advocate.

## The pursuit of appropriate human reliance on AI

Discovering the conditions that encourage effective collaboration between humans and artificial intelligence is essential, as current policies rely heavily on it as a safeguard (de Miguel et al., 2020; Green, 2022; Lazcoz & de Hert, 2023). Effective collaboration would imply neither an over-reliance on AI (Logg et al., 2019) nor an aversion to this technology (Burton et al., 2020; Daschner & Obermaier, 2022; Dietvorst et al., 2015). In such collaboration, humans should be able to appropriately calibrate their reliance on the AI based on its accuracy (Cabitza et al., 2021; Harbarth et al., 2024; Reverberi et al., 2022). If regulation mandates human oversight to mitigate the risks of AI use, these legal requirements should also define conditions and protocols to promote effective collaboration (Laux, 2023). However, current research cannot yet provide a clear picture of what these conditions and protocols would be.

It has been suggested that Explainable AI (XAI), which involves providing users with comprehensive explanations on how the AI arrived at a particular decision, could mitigate human over-reliance on AI advice (Cabitza, Campaganer, Natali et al., 2023; Famiglini et al., 2024; Lai & Tan, 2019; Pierce et al., 2022). However, research into Explainable AI has yielded mixed results, with some studies confirming the benefits of XAI and others suggesting that it not only fails to reduce people’s over-reliance on AI but actually increases it (Buçinca et al., 2021; Cabitza et al., 2023; Jabbour et al., 2023; Ostinelli et al., 2024; Vered et al., 2023). A recent systematic review and meta-analysis even found that the presence, or absence, of explanations did not significantly affect the performance of the human-AI team (Vaccaro et al., 2024).

A more direct and simple intervention, explicitly informing users of system errors, might increase their verification of AI suggestions, but even this strategy has not been clearly shown to protect the quality of individuals’ decisions from the influence of incorrect AI advice (Kupfer et al., 2023; Parasuraman & Manzey, 2010). Interestingly, a negative first impression of AI performance, for example, seeing the AI fail from the beginning, has demonstrated to reduce user’s tendency to follow AI advice in some studies, as individuals became less optimistic about the model’s reliability (Nourani et al., 2021; Vered et al., 2023). The downside is that a negative first impression could also lead to excessive reluctance to use AI (Dietvorst et al., 2015).

We have described key research findings on human-AI collaboration to illustrate that the optimal conditions for successful human monitoring of AI outcomes are still not well established (Parasuraman & Manzey, 2010; Vaccaro et al., 2024). Artificial intelligence has the potential to address some of the most significant societal challenges. However, to fully reap the benefits of this technology, it is essential to develop strategies to ensure protection against risks associated with its applications. Moreover, this need is even more pressing considering that, as we will explain below, the risk of AI having a negative impact on human behaviour extends beyond the immediate moment when an individual follows an erroneous AI recommendation for a particular decision.

## The risk of bias transmission from AI to humans

Interaction with AI models can significantly alter people’s decision-making strategies in a persistent way (Kwong et al., 2024; Vicente & Matute, 2023). Kwong et al.’s (2024) case study reported evidence of an unintentional change in a group of clinicians’ decision-making after repeated exposure to the predictions of an AI model. Kwong et al (2024) believe that the clinicians intuitively guessed and acquired the criteria on which the AI relied to make its predictions. Thus, people can learn from their interactions with machines, even if they are not fully aware of it (Glickman & Sharot, 2024). Moreover, if the AI model is flawed, individuals who have interacted with it repeatedly are at risk of inheriting its biases. In a previous study by Vicente and Matute (2023) participants who were assisted by a biased AI mimicked the bias of the model when making decisions on their own, even time after their collaboration with the AI had ceased. In that study, participants performed a binary classification task that simulated a medical diagnosis. The task required visual discrimination according to clear classification criteria, which participants were able to perform well without any assistance. Similarly, AI errors were noticeable. Thus, it would have been easy for participants to avoid excessive dependence on the AI’s systematically erroneous suggestions. However, the biased AI not only led participants to make mistakes they would have avoided without its assistance, but also propagated its biases into these individuals’ future decisions (Vicente & Matute, 2023). There is a high risk that when humans interact with AI, their existing biases will be reinforced or they will acquire new ones (Glickman & Sharot, 2024, 2025).

As mentioned above, it is still unclear which strategies might be most effective in preventing people from following erroneous AI advice. The research gap is even larger in the case of approaches to protect humans from acquiring biases introduced by AI. In this paper, we present the results of three experiments that tested simple interventions designed to encourage more critical and thoughtful human oversight of AI outcomes. Our primary focus was on evaluating the potential of these strategies to prevent humans from acquiring biases from AI, rather than merely mitigating the effects of systematic AI errors on human decision-making.

## Protecting humans from inheriting AI bias: overview of our experiments

Raising users’ awareness of the risk of error or bias in AI systems could be a simple but effective way to encourage a more critical assessment of AI performance. According to the AI Act, users need to understand the reliability and risk of bias of any AI-based tool that will support their decisions, particularly in high-impact contexts such as health or justice (Council of the European Union, 2024). What might be the most effective ways to present AI information to reduce the negative effect of its incorrect recommendations? In three different experiments, we tested two interventions aimed at mitigating the impact of a biased AI on participants’ decisions in a setting that simulated a medical diagnosis: participants had to observe and classify tissue samples from different patients (i.e. a simulated high-impact healthcare context). Due to ethical considerations, as well as to prevent the influence of prior knowledge and beliefs on the experiments’ results, the clinical context, the classification task, the artificial intelligence system, the tissue sample images and the syndromes used in the experiments, were all fictitious.

In Experiment 1 we aimed to test whether the framing of the information about the particular AI with which participants were going to work could influence their interpretation of that information. In other words, we wanted to know whether emphasising the percentage of incorrect recommendations versus the percentage of correct recommendations when reporting AI accuracy would lead to differences in participants’ tendency to follow the model’s suggestions, even though identical information about the reliability of the AI would be provided in both scenarios.

In Experiment 2, we tested whether warning participants about potential errors and biases in AI in general would be sufficient to prevent them from following erroneous AI recommendations, as opposed to the more specific message used in the previous experiment, which focused on the percentage of correct and incorrect AI recommendations. Raising participants’ awareness of potential AI biases could be achieved with a simple warning message. Similar interventions have been effective in promoting exhaustive search information strategies to counteract the influence of biased search engine results (Epstein & Robertson, 2017) and in protecting memory accuracy from misinformation (Karanian et al., 2020).

In addition, the design of Experiment 2 allowed us to explore whether the way in which information about AI errors influence behaviour would be affected by the type of AI mistake made: false positives versus false negatives. In the healthcare context in which we set our experimental task, a false-positive or false-negative error in recommending a diagnosis could have different implications. This could result in participants being more or less inclined to accept or reject the system’s support depending on the type of error.

The procedure for Experiment 3 was similar to that for Experiment 2, except that an additional control group was included. This group was informed about the risks of errors in AI’s outputs, but did not receive any AI support during the classification task. This unassisted group classified independently, enabling us to explore and eliminate the possibility of pre-existing response tendencies or biases that could have influenced the results. Furthermore, Experiment 3 helped to shed light on the potential influence of the type of error.

In summary, in a series of three experiments we empirically investigated whether a warning message emphasising the risk of error in AI recommendations could motivate participants to critically evaluate the recommendations of this agent, thereby minimising the influence of AI errors on their decisions and the risk of acquiring AI biases. We also explored the role that the framing of information (i.e., 20%-wrong vs. 80%-correct regarding AI accuracy in Experiment 1 and false positive vs. false negative AI bias in Experiment 2) may play in the tendency of participants to accept or reject AI suggestions and, consequently, in the effectiveness of the intervention (i.e., the error warning message). Table 1 summarises the experimental design of the three experiments.

**Table 1:** Design summary of the three experiments

Experiment	Group	Version	Error	*n*	Classification task			
					Phase 1	Trials	Phase 2	Trials
1	20%-wrong	B1/B2	FP/FN	64	AI-assisted	60	Unassisted	25
	80%-correct	B1/B2	FP/FN	52				
	No-info	B1/B2	FP/FN	59				
2	Info-error	B1/B2	FN/FP	100	AI-assisted	60	Unassisted	25
	No-info	B1/B2	FN/FP	98				
3	Info-error AI	B1/B2	FN/FP	81	AI-assisted	60	Unassisted	25
	No info AI	B1/B2	FN/FP	84				
	Info-error unassisted	B1/B2	FN/FP	78	Unassisted	60	Unassisted	25

The *Version* column specifies the two versions of the task instructions: in B1, participants were informed that a greater proportion of dark-coloured cells in the tissue sample indicated that the patient was affected by the disease, whereas in B2, a greater proportion of light-coloured cells indicated the presence of the disease. The *Error* column reports the type of errors associated with each version: FP, means false positive, and FN, false negative. This nomenclature refers to the two types of errors that the AI (and participants, if they followed the AI recommendation) could make when classifying tissue samples. The slash (/) indicates that the two versions of the instructions were counterbalanced; therefore, within each group, participants were randomly assigned to one of the two versions (B1/B2), so that they were randomly exposed to an AI system that systematically produced either false positives or false negatives. *n* is the number of final participants in each group. The two columns labelled *Trials* indicate the number of tissue sample images used in Phase 1 and Phase 2 of the classification task. As each tissue sample was shown separately on the screen, participants viewed one image per trial and the total number of images equals the total number of trials per phase

## Experiment 1: The Effect of Information Framing

In Experiment 1, one group of participants was informed of the percentage of errors made by the AI system they were going to interact with. A second group received information about the percentage of correct recommendations made by the AI system. The control group received no information about the accuracy of the AI system assisting them during the task.

When making decisions, individuals may become biased towards a particular choice depending on how information about the available options is presented (Kahneman, 2003, 2012; Levin et al., 1998). Research in psychology has repeatedly shown that different descriptions of the same choice problem can lead to different preferences and judgements (Kahneman, 2012; McNeil et al., 1982; Tversky & Kahneman, 1981). This effect has been demonstrated in many different contexts, including medical and clinical decisions, consumer choices, responses to social dilemmas and bargaining behaviours (Kahneman, 2012; Levin et al., 1998).

One well-established form of framing manipulation is attribute framing, whereby a specific characteristic of an object or event is emphasised. With this type of framing, presenting the same key information in either a positive or negative way can result in people making different judgements and forming different preferences. For instance, Levin et al. (1988) observed that participants rated a medical treatment as more effective and were more willing to recommend it to others when its outcome was described as having a “50% success rate” rather than a “50% failure rate”, despite the objective equivalence of the two descriptions.

Building on this evidence, subsequent research has shown that attribute framing also affects reliance on automated systems. Lacson et al. (2005) found that the way in which information about the reliability of a diagnostic aid was presented (80% correct vs. 20% incorrect) influenced people’s use of automation. Consequently, the importance of framing effects in AI-supported decision-making is increasingly recognised. The way in which the AI is presented shapes user expectations (Liao & Sundar, 2021; Pataranutaporn et al., 2023). Kim and Song (2020, 2023) examined whether the presentation of information about AI performance influences user trust and reliance. They compared three framing conditions: no performance information; negative framing (“the AI has a 20% error rate”); and positive framing (“the AI has 80% accuracy”). The results showed that, relative to the no-information condition, participants reported lower trust and were less likely to accept the AI’s recommendations when performance information was provided, regardless of whether it was framed positively or negatively. These findings suggest that providing performance information could make users more reflective and critical of AI recommendations. However, Kim and Song (2020, 2023) did not find any differences between positive and negative framing conditions.

We extend these earlier findings by examining the effects of information framing on simulated health-related decision-making, a context that is closer to real-world settings and involves higher stakes than those studied by Lacson et al. (2005) and Kim and Song (2023) (i.e., detecting a signal letter among noise letters and the Desert Survival Problem, respectively). Investigating the framing of AI-related information is particularly important in high-stakes domains such as clinical diagnosis, where even experts are susceptible to framing effects. For example, physicians are more likely to choose a surgical procedure when outcomes are framed positively in terms of survival rates rather than negatively in terms of mortality rates (Marteau, 1989; McGettigan et al., 1999; McNeil et al., 1982) Accordingly, the way information about an AI-based system’s accuracy is framed may substantially influence users’ perceptions of risk and their reliance on the system’s recommendations.

In addition, in our study the decision aid was a simulated biased AI system. We expected that the framing of accuracy information would influence participants’ tendency to accept the biased AI advice. Importantly, our study extends prior research by specifically testing whether the framing of accuracy information influences the likelihood of people acquiring AI bias. We predicted that information highlighting the AI’s errors would make participants more critical of its recommendations, thereby also reducing the risk of bias transmission.

## Method

### Participants

The final sample comprised 175 university students (mean age 18.5 years old, *SD* = 0.83). Among them, 82.3% self-identified as female, 16.6% as male, and 2% as non-binary. Initially, 179 students took part in the experiment but data from four participants were excluded following the preregistered data selection criteria described in the Procedure section below.

Participants were randomly distributed between the groups 20%-wrong (*n* = 64), 80%-correct (*n* = 52) and no-info (*n* = 59). A posteriori sensitivity analysis revealed that with this sample size, we obtained a 0.80 power to detect a small-sized effect of *f* = 0.10 or higher for a 3 (group) × 3 (block) mixed ANOVA.

### Procedure

The experimental task, created through Qualtrics (https://www.qualtrics.com/), was based on the procedure described by Vicente and Matute (2023). Participants had to imagine that they were clinicians and had to detect patients affected by a rare disease called Lyndsay Syndrome. Their task was to observe different tissue samples extracted from different patients and classify them as “Positive”, if the tissue sample showed evidence of being affected by the syndrome, or “Negative” if the evidence indicated that it was not affected. Each fictitious tissue sample had cells of two colours, dark pink and light yellow, in varying proportions, but one of the colours was presented in a greater quantity. The different proportions of dark and light-coloured cells for different stimuli were 80/20, 70/30, 60/40, 40/60, 30/70, and 20/80. Thus, this characteristic defined whether the tissue sample was affected or was not affected by the Lindsay Syndrome. The association of a greater number of dark or light cells with the positive and negative categories was counterbalanced, resulting in two possible sets of instructions to which participants were randomly assigned. The two possible classification criteria that participants followed are described in Table 2.

**Table 2:** Criteria for the categorization of tissue samples as positive or negative based on the predominant colour in the sample

Instructions	A greater number of dark-coloured cells	A greater number of light-coloured cells
B1	Positive	Negative
B2	Negative	Positive

The two possible combinations of the predominant dark or light colour in the tissue sample with the positive/negative categories resulted in two different sets of instructions B1 and B2. The assignment to B1 or B2 instructions was randomly decided for each participant

At the beginning of the experiment, participants had to successfully classify a series of practice trials to demonstrate that they had understood the experimental instructions and classification criteria. In this practice phase, participants categorised six tissue samples with different dark/light colour proportions (i.e., 80/20, 70/30, 60/40, 40/60, 30/70, and 20/80), each of which was presented twice. That is, the practice consisted of two blocks of the same six stimuli, presented one per trial, resulting in 12 trials in total. The first six samples were presented in order of difficulty, with participants receiving feedback on whether their answer was correct. If they made a mistake, they had to correct it before moving on to the next trial. In the second set of trials, the six samples were presented in random order. If participants did not get five correct classifications out of six trials in the second block, they had to repeat the instructions and all the practice trials. Data from participants who did not reach the threshold of five correct classifications out of six trials after repeating the practice phase were excluded from the analyses.

Once the practice trials were completed, participants were prepared to perform the tissue sample classification task. To this end, they were randomly assigned to one of three groups: no-info, 20%-wrong and 80%-correct. The only difference between these groups was the information given about the AI system at the beginning of the classification task. The no-info group received a brief general indication about the AI that would offer them recommendations when classifying tissue samples: “This diagnostic assistance system based on artificial intelligence offers recommendations to help doctors to classify tissue samples”. The other two groups received the same general explanation with an additional statement about the accuracy rate. For the 20%-wrong group, it was: “It is important to note that 20% of the recommendations made by the AI are wrong”. Conversely, for the 80%-correct group, the information was: “It is important to note that 80% of the recommendations made by the AI are correct”. An accuracy rate of 80% and an error rate of 20% are realistic metrics for the simulated AI in our study. These accuracy rates are consistent with those of sophisticated AI models in various diagnostic imaging tasks, which range from 70 to 99% (Bai et al., 2024; Deng et al., 2024; Jeong et al., 2025; Liu et al., 2019; Mäenpää & Korja, 2024; Sabeghi et al., 2024; Thong et al., 2023; Wang, 2024). Furthermore, this manipulation is in line with previous studies that have examined how the framing of accuracy information affects the adoption of automation (Kim & Song, 2020, 2023; Lacson et al., 2005).

**Fig. 1:**
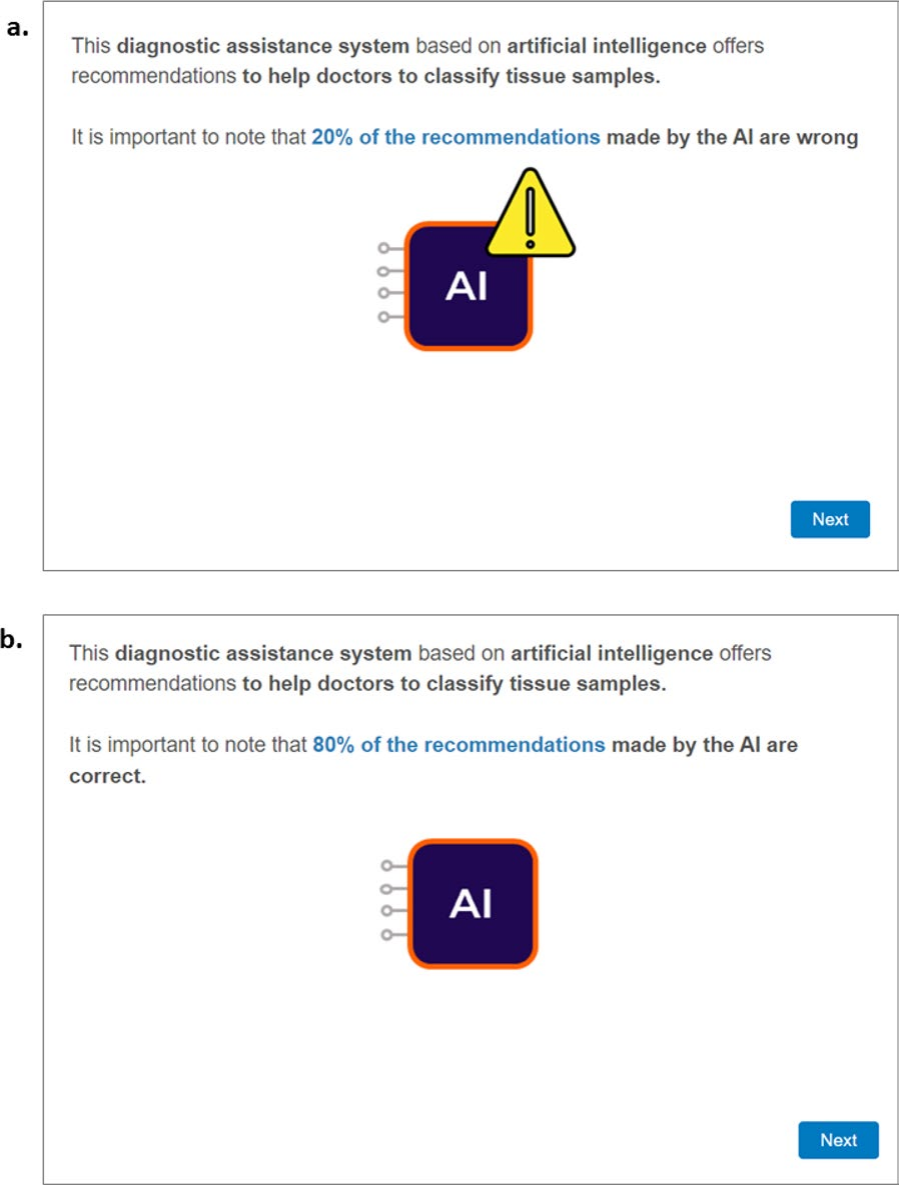
Screenshots illustrating the information about the AI system of the experiment presented to the 20%-wrong and 80%-correct groups in Experiment 1. **a** illustrates the AI error information that participants in the 20%-wrong group read at the beginning of the task while **b** displays the AI accuracy information presented to participants in the 80%-correct group. Please note that participants in the no-info condition did not receive any indication of AI accuracy or error information; they were only instructed that the diagnostic assistance system based on AI offered recommendations to help doctors classify the tissue samples. The original instructions were written in Spanish as we recruited Spanish-speaking students to take part in Experiment 1

Figure 1 depicts screenshots exemplifying the AI information showed to the 20%-wrong and 80%-groups. After reading these messages, the three groups completed exactly the same tissue sample classification task according to the instructions received (see Tables 1 and 2) and answered the same post-experimental questions.

The classification task was divided into two phases, identical for all the groups: Phase 1 which was AI-assisted and Phase 2 which was unassisted. In Phase 1, the hypothetical AI system provided participants with recommendations on how to classify tissue samples. The recommendations were in the form of labels placed above the tissue sample, either orange with the text “POSITIVE + ” or blue with the text “NEGATIVE –”. In each trial, the tissue sample to classify and the AI recommendation were presented at the same time, allowing participants to compare both pieces of information so that they could follow or ignore the AI advice as they wished.

**Fig. 2:**
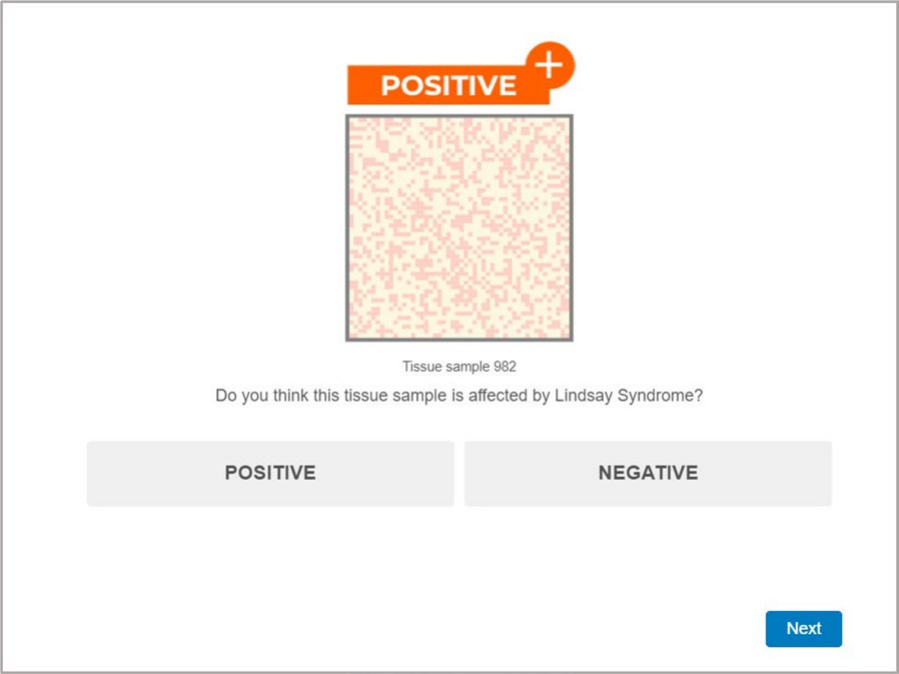
Screenshot illustrating a biased AI recommendation for a sample with 40% dark-Ccloured cells and 60% light-coloured cells. The 40/60 samples should have been classified as “Negative” following the instructions B1, as these samples were characterised by a higher proportion of light-coloured cells, however the AI consistently recommended classifying the 40/60 as “Positive” in B1. The AI bias was reversed for the B2 instructions

Phase 1 of the classification task consisted of 60 trials in total, with ten tissue samples (i.e., ten trials) of each of the dark/light cells proportions, that is 80/20, 70/30, 60/40, 40/60, 30/70 and 20/80. If participants failed to correctly classify more than half of the trials in the first phase of the classification task (i.e. more than 30 trials out of 60), their data was also excluded from the analysis. During Phase 1, the AI model made incorrect recommendations in 10 out of 60 trials, which means that the 16.7% of recommendations made by the AI were erroneous, while the recommendations made by the AI for the remaining 50 out of 60 trials were correct, which results in a 83.3% of correct recommendations. The peculiarity of this AI model was that it always offered a systematically erroneous recommendation for the ten stimuli with a dark/light cells ratio of 40/60. That is, the fictitious AI was biased (see Fig. 2).

Please note that the description of the AI system’s accuracy presented to participants does not completely match its actual performance. We decided to report the accuracy percentages as rounded numbers to simplify the interpretation of the instructions. For instance, 16.7% was rounded to 20%, and 83.3% was rounded to 80%. This number, 80%, is often perceived as high accuracy, leading users to consider models with accuracy above this threshold as reliable (Yin et al., 2019; Yu et al., 2019). Likewise, a 20% error rate clearly falls within the low accuracy range (Chong et al., 2023; Yu et al., 2019) meaning that a percentage of 16.7% (which is only 3.3 points below that threshold) would hold a similar interpretation. We selected the 20% and 80% percentages for ease of understanding.

**Fig. 3:**
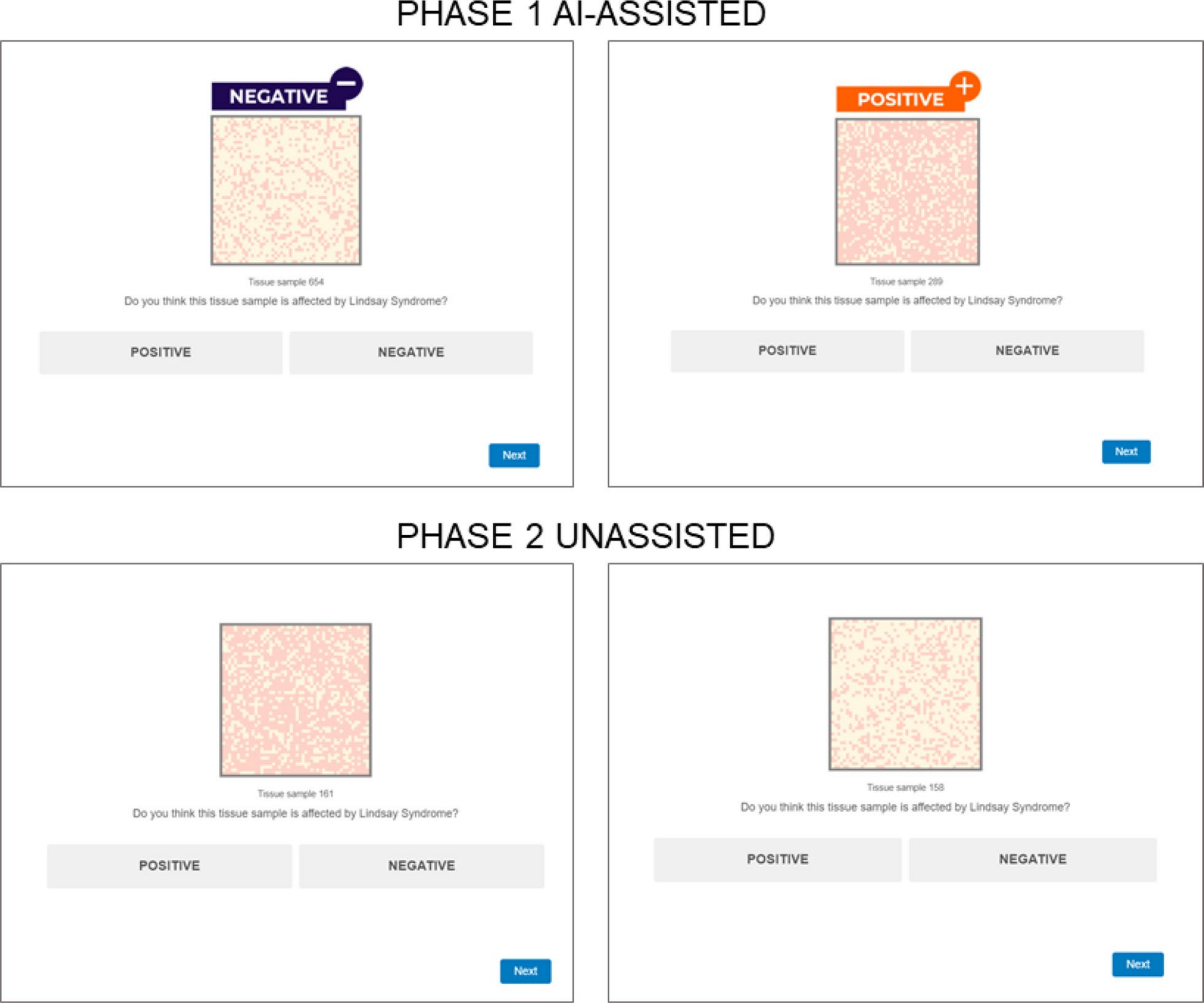
Screenshots showing examples of trials from Phase 1 and Phase 2 of the classification task. *S*timuli were adapted from “Are the symptoms really remitting? How the subjective interpretation of outcomes can produce an illusion of causality” by Blanco, F., Moreno-Fernández, M. M., and Matute, H. *Judgment and Decision Making,* 15, pp. 575 (2020). CC BY 3.0. The stimuli used in the experimental task are openly available at the Open Science Framework (OSF) at https://osf.io/ukehm/

In the second phase of the classification task, participants were informed that the AI had been disconnected, so they would have to perform the classification task without assistance. During Phase 2, only the tissue sample to classify was presented to participants in each trial, without any additional information (some examples of AI-assisted Phase 1 and non-assisted Phase 2 trials are shown in Fig. 3). This second phase of the task consisted of 25 trials in total, with five stimuli of each of the 70/30, 60/40, 40/60, and 30/70 proportions (80/20 and 20/80 proportions were not included in this phase of the task for being considered too easy). Importantly, Phase 2 was also characterised by the appearance of five trials of new ambiguous stimuli with a dark/light cell ratio of 50/50.

During Phase 1 and Phase 2 of the experiment, the order of the tissue samples (or stimuli) was randomly assigned to the sequence of 60 and 25 trials, respectively. The 40/60 samples did not appear between the first ten trials in Phase 1 nor between the first five trials in Phase 2. These 40/60 samples appeared in the sequence of the next trials randomly intermixed with the samples of other proportions for both phases. The purpose of this manipulation was to prevent participants from developing a negative first impression of the AI model, which could have resulted in a loss of trust and its effect could have been confounded with the effect of our experimental manipulation: specific information reporting AI accuracy or AI error rate.

After the end of the classification task, participants from the three groups were asked to estimate the percentage of error they perceived in the recommendations made by the AI over the total of trials of the classification task. They answered in a scale from 0 to 100%. We considered that a continuous scale was a sensitive measure to capture participant’s perceived accuracy of the AI system. After, we required them to indicate on a scale from 1 (*nothing*) to 9 *(totally*) to what extent they considered the AI of the experiment to be helpful, to what extent they followed the AI recommendations and which level of trust they placed in artificial intelligence applied to healthcare. These questions were presented always in the same order to participants.

In Experiment 1, the main dependent variable was the participants’ error rates in the 40/60 samples of the classification task, which were the samples in which the AI provided its incorrect recommendation during Phase 1. In order to analyse changes in participants’ behaviour throughout the classification task we divided the total number of 40/60 trials of the task into three blocks of five trials each. The ten 40/60 trials in Phase 1 were divided into two blocks of five trials each, while the five 40/60 trials in Phase 2 formed the third block. We made these blocks of trials purely for statistical analysis purposes, and this did not affect the procedure followed by the participants in any way.

The mean ratio of classification errors in 40/60 samples in Phase 1 was a measure of the influence of biased AI recommendations on participants’ decisions. In Phase 2, participants were required to classify tissue samples, as they did in Phase 1, but without recommendations. This allowed us to analyse how prior interaction with the biased AI system affected participants’ future decision-making in a context without AI, specifically for 40/60 samples. During Phase 2, we expected participants to classify 40/60 samples in the same category as the biased AI did in the previous phase, similar to Vicente and Matute’s (2023) results. We interpret this behaviour as an inheritance of AI bias. Thus, the ratio of misclassification of 40/60 samples in Phase 2 was a measure of participants’ acquisition of AI bias.

A second dependent variable in Experiment 1 was the number of biased classifications of the five 50/50 ambiguous stimuli in Phase 2. This variable was the number of times a participant classified the five 50/50 stimuli in the same direction as the biased recommendations of the AI indicated for the 40/60 samples in the previous phase. In the absence of objective information, we expected participants to use AI biased recommendations from the previous phase as a reference to classify ambiguous 50/50 samples into one of the two possible categories (Positive or Negative), given its similarity with 40/60 samples. We consider these biased classifications of 50/50 samples an index of bias generalisation. In summary, Phase 2 tested the inheritance of AI bias and its generalisation to new and ambiguous stimuli.

Our main hypothesis for the Experiment 1 was that emphasising the AI error rate would make participants less prone to uncritically accept AI recommendations than emphasising the rate of correct AI results or not reporting any AI accuracy or error data. As a consequence, highlighting the 20% error rate would protect participants from inheriting AI bias and from its generalisation. Then, we expected that participants from the 20%-wrong group would make fewer errors in the 40/60 samples classification in both Phase 1 and Phase 2 of the task than the group of participants that did not receive any information about the AI error rate. This no-info group would tend to frequently misclassify 40/60 samples in the AI-assisted (Phase 1) and unassisted phase (Phase 2) of the task influenced by the AI bias. Our predictions for the 80%-correct group were not as clear as those for the other two groups. This group would probably make fewer errors than the non-informed group, but we did not expect their performance to improve as much as the group informed about the 20% error rate in AI recommendations. In addition, we expected the 20%-wrong to classify randomly and unbiasedly the ambiguous 50/50 stimuli while the no-info group would tend to classify the new ambiguous stimuli of Phase 2 in the same direction as the AI bias for 40/60 stimuli. Thus, the no-info group would replicate the same inheritance and generalisation of AI bias observed in Vicente and Matute (2023), whereas a mitigation of this effect would be observed for the 20%-wrong group.

The experimental design, conditions, main hypothesis, dependent variables, sample size, participant exclusion criteria and statistical tests for Experiment 1 were preregistered on Aspredicted.org before data collection and analysis (see https://aspredicted.org/xg7h5.pdf).

### Results and discussion

**Fig. 4:**
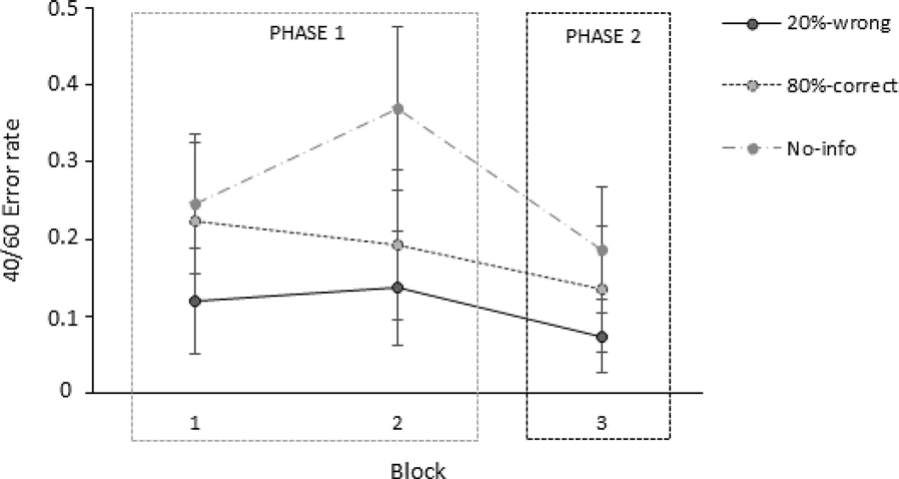
Participants’ mean error rates on each of the three blocks of 40/60 trials of the classification task for each group in Experiment 1. Error bars depict the 95% confidence intervals for the means

Figure 4 depicts the mean error rates of each group per block of five 40/60 trials of the classification task. As can be observed in Fig. 4, the 20%-wrong group made fewer errors than the other two groups in the classification of 40/60 samples while the no-info group showed the highest number of errors, as we expected. Furthermore, the task performance of the no-info group followed an unstable trend, with an increase in 40/60 errors between Block 1 and Block 2, which were AI assisted, and a marked reduction in Block 3, in which the AI was no longer present. Conversely, the 20%-wrong and 80%-correct groups showed a more constant behaviour across the three blocks of trials.

A mixed ANOVA 3 (group: 20%-wrong, 80%-correct, no-info) × 3 (block: block 1, block 2, block 3) on the mean error rates in the classification of 40/60 samples showed a main effect of group, *F*(2,172) = 3.82, *p* =.024, *η*^2^_*p*_ =.043, a main effect of block, *F*(2,344) = 14.27, *p* <.001, *η*^2^_*p*_ =.077, and a Group x Block interaction* F*(4,344) = 4.05, *p* =.003, *η*^2^_*p*_ =.045. Tukey post hoc comparisons showed a significant difference in the mean rate of 40/60 misclassifications between the 20%-wrong and no-info groups in Block 2, *t*(172) = 3.41, *p*_*tukey*_ =.022 but no significant differences were found between the 80%-correct and no-info groups, *t*(172) = 2.45, *p*_*tukey*_ =.262 or between the 20%-wrong and 80%-correct groups, *t*(172) =  − 0.80, *p*_tukey_ =.997, for this second block of trials. There were no differences between the groups in Block 1 or Block 3. On the other hand, post hoc comparisons also aligned with the trend observed in Fig. 4 for the no-info group with a significant increase of 40/60 errors between Block 1 and Block 2, *t*(172) =  − 4.68, *p*_tukey_ <.001, and a significant decrease between Block 2 and Block 3, *t*(172) = 4.86, *p*_tukey_ <.001. A similar tendency was not observed for either of the two other experimental groups. The warning that 20% of the AI recommendations were incorrect prevented participants from making errors in Phase 1 and Phase 2, at least in comparison with the no-information condition. However, the significant differences between the 20%-wrong group and the no-info group only arose in the second block of trials of Phase 1, given the increase in errors in the no-info group and the more constant response tendency in the 20%-group. It seems that throughout Phase 1 of the task, non-informed participants increasingly adhered to AI suggestions while the 20%-wrong remained consistently cautious.

We also conducted a one-way analysis of variance to explore between-group differences in the tendency to categorise ambiguous stimuli 50/50 in the same direction as the AI bias in Phase 2 (when participants no longer had AI recommendations). The mean rate of biased classifications was 0.54 (*SD* = 0.43) for the no-info group, 0.45 (*SD* = 0.42) for the 20%-wrong group, and 0.46 (*SD* = 0.35) for the 80%-correct group. The ANOVA revealed that there were no statistically significant differences in the tendency to categorise ambiguous stimuli as a function of group,* F*(2,172) =.935, *p* =.395, *η*^2^_*p*_ =.011. That is, prior information about the risk of error in AI recommendation did not introduce differences in participants’ tendency to generalise the AI bias to new ambiguous stimuli.

**Fig. 5:**
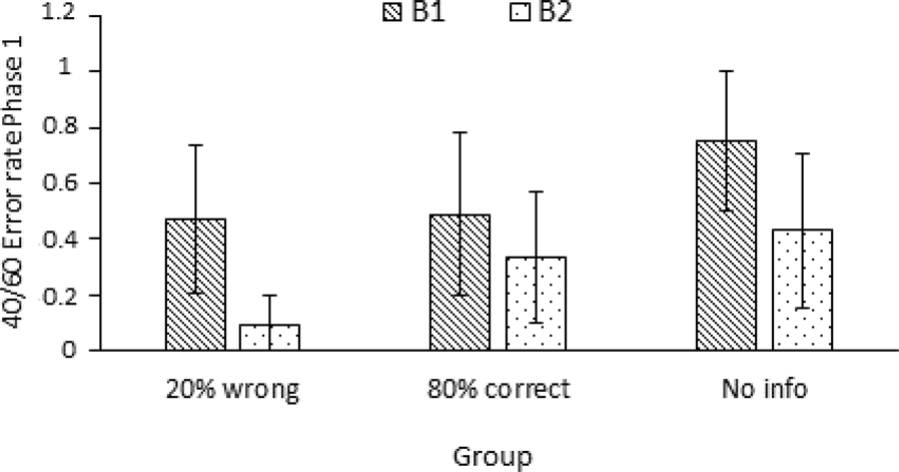
Participants’ mean error rates on the ten 40/60 trials of Phase 1 of as a function of group (20%-wrong, 80%-correct, No-info) and instructions (B1, B2) in Experiment 1. Error bars depict the 95% confidence intervals for the means

Next, we decided to examine whether the set of instructions participants were randomly assigned to, B1 or B2, had any effect on their behaviour in Experiment 1 (Fig. 5). A two-way ANOVA 3 (group: 20%-wrong, 80%-correct, no-info) × 2 (instructions: B1, B2) on the mean ratio of classification errors participants made on 40/60 trials in Phase 1 revealed a main effect of instructions, *F*(1,169) = 9.061, *p* =.003, *η*^2^_*p*_ =.051, but not a main effect of group, *F*(1,169) = 2.738, *p* =.068, *η*^2^_*p*_ =.031*.*There was not a Group × Instructions interaction, *F*(1,169) = 0.521, *p* =.595, *η*^2^_*p*_ =.006. The same model of ANOVA on the 40/60 classification errors in Phase 2 did not find an effect of group, *F*(1,169) = 2.071, *p* =.129, *η*^2^_*p*_ =.024, an effect of instructions, *F*(1,169) = 3.817, *p* =.052, *η*^2^_*p*_ =.022, neither an interaction between the two factors *F*(1,169) = 0.213, *p* =.808, *η*^2^_*p*_ =.003.

Participants in Experiment 1 were also asked to estimate the perceived error rate in the AI recommendations on a scale from 0 to 100. The 20%-wrong group estimated an AI error rate of 27.3% (*SD* = 14.1), while the 80%-correct group estimated a higher error rate of 35% (*SD* = 23.5). Interestingly, the no-info group estimated the most accurate error rate, 22.3% (*SD* = 13.4), which was close to the actual error rate of the fictitious AI model used in this experiment. Despite being quite accurate estimating the error rate of the AI, the no-info group still was influenced by its biased recommendations.

In Experiment 1, the participants who tended to accept the biased AI advice more during Phase 1 of the classification task and, as a consequence, made more errors in the 40/60 samples classification, were also the participants who reported that AI was more helpful, *r* =.46, *p* <.001, followed more the AI advice to classify the samples, *r* =.58, *p* <.001, and trusted more in AI applied to the health domain, *r* =.37, *p* <.001.

In sum, participants informed of the 20%-wrong AI’s recommendations were less likely to accept system’s erroneous advice, as demonstrated by the lower mean number of errors in 40/60 samples as compared to those who were informed of the 80% correct recommendations or those who received no information at all. We observed no differences in error rates between groups when classifying samples with other dark/light cells ratios (excluding the 40/60 samples) as shown in Table 3. Information about the AI’s percentage of correct or incorrect classifications did not appear to influence participants’ performance when the AI provided correct recommendations.

**Table 3:** Mean error rates (and standard deviations) for all stimuli of different proportions of dark/light cells used in the classification task in Experiment 1

Group	Phase 1						Phase 2			
	80/20	70/30	60/40	40/60	30/70	20/80	70/30	60/40	40/60	30/70
20% wrong	0.00 (0.04)	0.01 (0.08)	0.07 (0.16)	0.13 (0.28)	0.00 (0.02)	0.00 (0.01)	0.01 (0.12)	0.05 (0.15)	0.07 (0.19)	0.00 (0.04)
80% correct	0.00 (0.03)	0.00 (0.03)	0.06 (0.11)	0.20 (0.34)	0.00 (0.01)	0.00 (0.00)	0.00 (0.00)	0.03 (0.11)	0.13 (0.30)	0.01 (0.11)
No info	0.00 (0.00)	0.00 (0.02)	0.05 (0.08)	0.29 (0.37)	0.00 (0.01)	0.00 (0.00)	0.00 (0.02)	0.03 (0.12)	0.19 (0.33)	0.01 (0.06)

The misclassification rate per stimulus class is the total number of classification errors made on stimuli of a given class divided by the total number of such stimuli in each phase of the experiment

Participants instructed on the B1 version of the classification criteria made more errors in the classification of 40/60 samples than participants that followed the B2 version of the instructions (recall that both versions were equivalent, only with a reversed assignment of the dark/light colours to the positive/negative categories). Although the instructions’ effect may have been confounded with the effect of our manipulation, we still found that the 20%-wrong group made fewer classification errors than the no-information group, even after controlling for the difference in participants’ behaviour introduced by criteria B1 and B2 (see Fig. 5). Therefore, we can conclude that the effect of the instructions does not compromise our main result.

The difference between B1 and B2 instructions in the mean number of errors in the 40/60 samples in Experiment 1 was unexpected, as it had not been previously observed in experiments using the same procedure (Vicente & Matute, 2023). We noted that following classification criteria B1, the biased recommendation made by the AI on 40/60 samples consisted of a positive label for a sample that should be classified as a negative. That is, the AI made a false positive error. However, in B2 the erroneous AI suggestion was negative when the 40/60 sample would be positive, meaning the AI made a false negative error. In the medical context of the experimental task, false positive errors (e.g., diagnosing the disease in a healthy patient) and false negative errors (e.g., failing to detect the disease in an affected patient) have distinct meanings and consequences. False negative errors could be perceived as more serious, for example if they result in patients not receiving necessary medical care, than false positives. Thus, the direction of the AI bias may have had different meanings for the participants, leading to differences in their behaviour. Given the intriguing results observed in Experiment 1, we decided to investigate the effect of the type of error made by the AI in the next experiment.

## Experiment 2: General Error Message and Type of AI Bias

Experiment 2 was designed to answer two questions derived from the results of the previous experiment. First, would a simpler and less specific message (compared to reporting the percentage of error in AI results) alerting participants to the possibility of error in AI recommendations be sufficient to achieve the same effect? We believe that a simpler message might be even more salient to participants, easier to process, and easier to remember. Second, we explored whether the type of systematic errors made by the AI (or AI type of bias), false positives versus false negatives, could have a different impact on the participants’ tendency to follow the AI’s advice. For instance, studies investigating the effect of automated aid systems in signal detection tasks have found that people adopt different strategies depending on whether the errors made by the signalling system are mainly misses or false alarms (Chancey et al., 2017; Dixon et al., 2007). By drawing some parallels between our procedure and the signal detection paradigm, we can conjecture that an AI system biased towards false negatives might elicit different responses from participants than an AI system biased towards false positives.

## Method

### Participants

We recruited 200 participants through the Prolific platform (https://www.prolific.com/), but data from two participants was lost due to technical problems. Thus, 198 participants formed the final sample (mean age = 30.6, *SD* = 8.99; 58.1% female, 41.9% male). Prolific is the recruitment platform that provides researchers with the highest quality data of those currently available (Douglas et al., 2023). We used the filters provided by Prolific to recruit participants who had not taken part in previous experiments from our laboratory and who were fluent in English. The reward for performing the task, which took about 14 min on average, was £1.29. Experiment 2 included four different conditions that resulted from a combination of two factors: error information (information on potential AI errors [info-error] and no information [no-info]) and type of error (false positive [FP] and false negative [FN]). Therefore, participants were randomly distributed between the conditions: info-error FP (*n* = 50), info-error FN (*n* = 50), no-info FP (*n* = 49) or no-info FN (*n* = 49). Sensitivity analysis revealed that with a sample size of 198 participants, we could detect an effect size of *f* = 0.20 with 80% power for a 2 (group: info-error, no-info) × 2 (type of error: false positive, false negative) ANOVA.

### Procedure

In Experiment 2, we used the same task and procedure as in Experiment 1, with two main differences. First, in the present experiment there were two different information conditions (general error information or no information), contrasting with the three groups of Experiment 1, which required a change in the instructions. The second main change in Experiment 2 was the bias of the simulated AI. The AI system in Experiment 2 consistently made an incorrect recommendation for the samples with a dark/light ratio of 60/40. In the previous experiment, the AI system made an incorrect recommendation for the 40/60 samples.

**Fig. 6:**
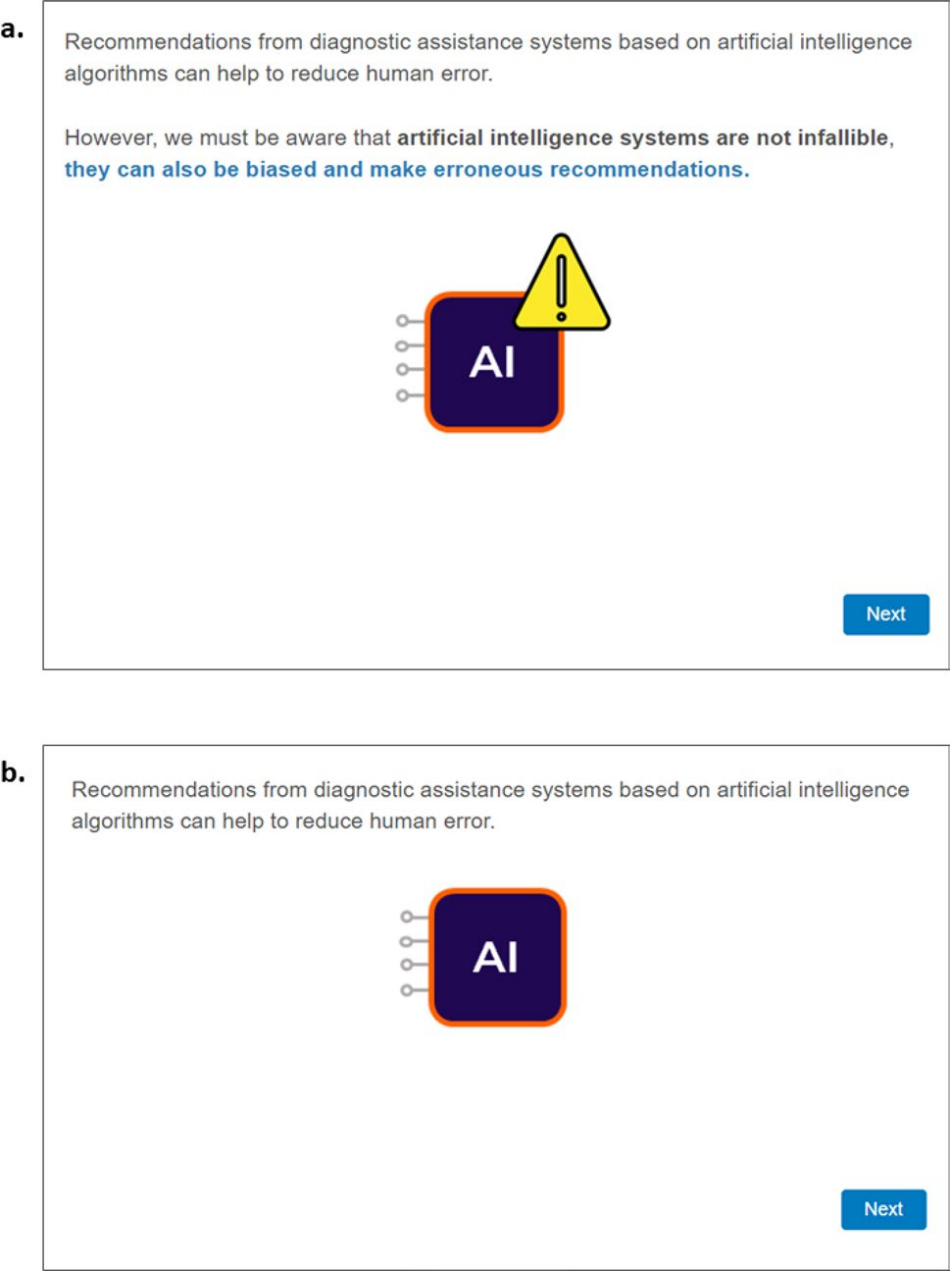
Screenshots showing the information about the AI system presented to **a** Error-info and **b** No-info groups in Experiment 2

In Experiment 2, we tested whether a simpler message, highlighting the possibility that the AI could make errors, could also be effective in dissuading participants from following AI erroneous suggestions. Therefore, the info-error group was shown the following warning message, presented on a separate screen: “Recommendations from diagnostic assistance systems based on artificial intelligence algorithms can help to reduce human error. However, we must be aware that artificial intelligence systems are not infallible, they can also be biased and make erroneous recommendations”. On the other hand, the no-info group was only shown the first sentence of the message: “Recommendations from diagnostic assistance systems based on artificial intelligence algorithms can help to reduce human error”. Thus, the no-info group was not made aware of biases or errors in AI systems. Figure 6 depicts screenshots of the instructions shown to the error-info and no-info groups.

As we introduced previously, in Experiment 2 we changed the AI bias from a systematic error in 40/60 samples, as in Experiment 1, to a systematic error in 60/40 samples. This change in the AI bias would allow us to obtain relevant information about the cause of the differences between B1 and B2 instructions observed in the previous experiment. For example, if such differences were caused by a perceptual factor associated with 40/60 samples, the differences would not appear when the bias was changed to 60/40 samples. However, if such differences between B1 and B2 were related to a fundamental difference in each version of the instructions (i.e., false positive or false negative errors), we would find differences in Experiment 2, but with a reversal in the direction compared to the previous experiment: a higher number of errors in B2 than in B1. The shift of the AI bias from a systematic error in 40/60 samples in Experiment 1 to a systematic error in 60/40 samples in Experiment 2 reversed the association of the false positive and false negative errors with the two alternative classification criteria B1 and B2, as specified in Table 4.

**Table 4:** Correct classification in each of the two instructions B1 and B2, the recommendation made by the AI in each case, and different types of error in the dark/light coloured cell stimuli 40/60 in experiment 1 and 60/40 in experiment 2

Experiment	Stimulus	Instructions	Correct classification	AI recommendation	Type of error
1	40/60	B1	Negative	Positive	False positive
		B2	Positive	Negative	False-negative
2	60/40	B1	Positive	Negative	False-negative
		B2	Negative	Positive	False positive

Consequently, in Experiment 2 we analysed whether the type of error made by the AI system, false positive or false negative, influenced participants’ tendency to follow its recommendations. In Experiment 1, participants may have assigned different meanings or implications to each type of error, although this was only a tentative explanation at this stage. Therefore, we needed to gain more information about the potential role that the type of systematic error may play in the context of our experimental task.

In the previous experiment, classifying an uncertain 40/60 sample as positive, as the AI advised (i.e. making a false positive) in the set of instructions B1, could have been perceived as the least risky error (classifying as positive a healthy patient to confirm later that was a mistake). In contrast, in the B2 instructions, classifying a 40/60 tissue sample, that appeared positive, as negative (the category suggested by the AI, making a false negative error) would have serious implications (missing a patient affected by the disease and in need of medical attention), so it was better to classify it as positive (i.e. the correct category). Thus, this strategy would have implied contradicting an incorrect AI suggestion in B2, but following an incorrect AI suggestion in B1. Experiment 2 could help to understand the pattern of results of Experiment 1: if the two types of errors (false positive and false negative) have different meanings for the participants, we would expect an inversion in the direction of participants’ mistakes between Experiment 1 and Experiment 2. In the second experiment, participants would more frequently follow AI recommendations for 60/40 samples when the AI made a false positive error, which would be perceived as a low-risk decision, but this error would occur in B2 rather than in B1. Conversely, participants would tend to ignore the AI recommendation when the type of AI error was a false negative, a condition in which the consequences of an error could be more severe. Thus, participants in the false positive condition (associated with instructions B2 in Experiment 2) would make more errors when classifying the 60/40 samples, than participants in the false negative condition (associated with B1 instructions in Experiment 2). Thus, participants would always make a higher mean number of errors when the AI failed towards a false positive, than a false negative, independently of the association of each type of error with B1 or B2.

Our hypothesis for Experiment 2 was that both the type of error (false positive vs. false negative) and the general message warning about the presence of errors in the AI (error information vs. no information) would influence participants’ decision to follow the biased recommendations of the AI. We expected participants to be more reluctant to follow the recommendation made by the AI when the risk was a false negative, but more likely to accept its advice when the consequence was a false positive. Concerning the warning about potential AI errors, we predicted that this information would prevent participants from uncritically following AI erroneous advice. That is, participants informed about potential AI errors would make fewer errors than participants who did not receive such information. Consequently, considering the effect of the two factors (type of error and error information) at the same time, we expected the general message warning of AI biases to mitigate participants’ errors particularly when the incorrect recommendation by the AI was a false positive, which is when participants would otherwise be making more errors. Conversely, in the false negative condition, the message warning of errors in the AI results would have less room to improve individuals’ performance. At the end of the classification task, participants in Experiment 2 answered the same questions about AI accuracy and about their trust in AI as in Experiment 1.

The hypothesis, experimental design, groups, dependent variables, sample size and exclusion criteria for participants’ responses in Experiment 2 were preregistered on aspredicted.org (see https://aspredicted.org/yz79a.pdf). For clarity, any analyses that were not preregistered but were conducted for exploratory purposes will be labelled as such in the Results and Discussion section.

### Results and discussion

To test the hypothesis of Experiment 2, we conducted a mixed ANOVA with group (error information [info-error] vs. no information [no-info]) and type of AI error^1^ (false positive [FP] vs. false negative [FN]) as between-subjects factors, and block of trials (Blocks 1, 2, and 3) as a within-subjects factor. The dependent variable was the mean error rate in the 60/40 cases.

The ANOVA showed a significant main effect of group, *F*(1,194) = 7.95, *p* =.005, *η*^*2*^_*p*_ =.039. No main effect of type of AI error was observed, *F*(1,194) = 1.23, *p* =.269, *η*^*2*^_*p*_ =.006, nor was there a significant interaction between group and type of error, *F*(1,194) = 2.57, *p* =.111, *η*^*2*^_*p*_ =.013. There was no main effect of block, *F*(2, 388) = 2.652, *p* =.072, *η*^*2*^_*p*_ =.013, and no significant interactions involving block: Block x Group, *F*(2,388) = 0.468, *p* =.627, *η*^*2*^_*p*_ =.002, Block x Type of error, *F*(2, 388) = 0.23, *p* =.795, *η*^*2*^_*p*_ =.001, or the three-way interaction Block × Group × Type of error, *F* (2,388) = 0.334, *p* =.716, *η*^*2*^_*p*_ =.002.

Although the ANOVA did not reveal an interaction between the information provided about AI errors and the type of error, we conducted post hoc comparisons (Tukey correction) to further explore whether the type of error might partially explain the unexpected pattern of results observed in Experiment 1 as a function of the instructions version. This analysis was entirely exploratory and was not preregistered. Post hoc analysis revealed a significant difference between info-error FP and no-info FP, *t*(194) =  − 3.127, *p*_*tukey*_ =.011, and between info-error FN and no-info FP, *t*(194) =  − 2.778, *p*_*tukey*_ =.030. No other between-group comparisons were significant.

Table 5 presents participants’ mean error rates across all case types with varying dark/light cell proportions in the classification task. In Experiment 2, participants who were not informed about potential AI errors (no-info FP and no-info FN in the table) showed higher error rates in the false positive condition than in the false negative condition. This pattern may suggest a greater tendency to follow AI recommendations for the 60/40 samples in the false positive condition, whereas participants appeared less inclined to follow biased AI recommendations in the false negative condition. Notably, this pattern emerged in the absence of a general warning about potential AI errors.

**Table 5:** Mean error rates (and standard deviations) for all stimuli of different proportions of dark/light cells used in the classification task in experiment 2

Group	Phase 1						Phase 2			
	80/20	70/30	60/40	40/60	30/70	20/80	70/30	60/40	40/60	30/70
Info-error FP	0.00 (0.00)	0.01 (0.00)	0.15 (0.27)	0.09 (0.20)	0.01 (0.07)	0.00 (0.01)	0.02 (0.09)	0.14 (0.28)	0.10 (0.22)	0.01 (0.08)
Info-error FN	0.00 (0.05)	0.01 (0.07)	0.19 (0.34)	0.03 (0.09)	0.00 (0.03)	0.00 (0.01)	0.01 (0.06)	0.13 (0.26)	0.02 (0.07)	0.00 (0.03)
No info FP	0.00 (0.02)	0.00 (0.03)	0.35 (0.40)	0.10 (0.21)	0.00 (0.03)	0.00 (0.01	0.03 (0.13)	0.31 (0.37)	0.08 (0.19)	0.00 (0.00)
No info FN	0.00 (0.00)	0.00 (0.01)	0.24 (0.34)	0.02 (0.09)	0.00 (0.02)	0.00 (0.00)	0.01 (0.08)	0.24 (0.34)	0.00 (0.02)	0.00 (0.02)

The misclassification rate per stimulus class is the total number of classification errors made on stimuli of a given class divided by the total number of such stimuli in each phase of the experiment

The warning information may have had a limited impact in the false negative condition, possibly because participants were already responding more cautiously and making fewer errors. Conversely, raising participants’ awareness of potential errors in AI recommendations could be more effective in reducing compliance with AI suggestions in cases of false positive bias, where participants tend to adhere more to AI suggestions. However, these observations are based on exploratory descriptive analyses, and the mixed ANOVA did not reveal a significant effect of error type. Accordingly, our interpretations are tentative and should be considered in light of the exploratory nature of the analyses.

**Fig. 7:**
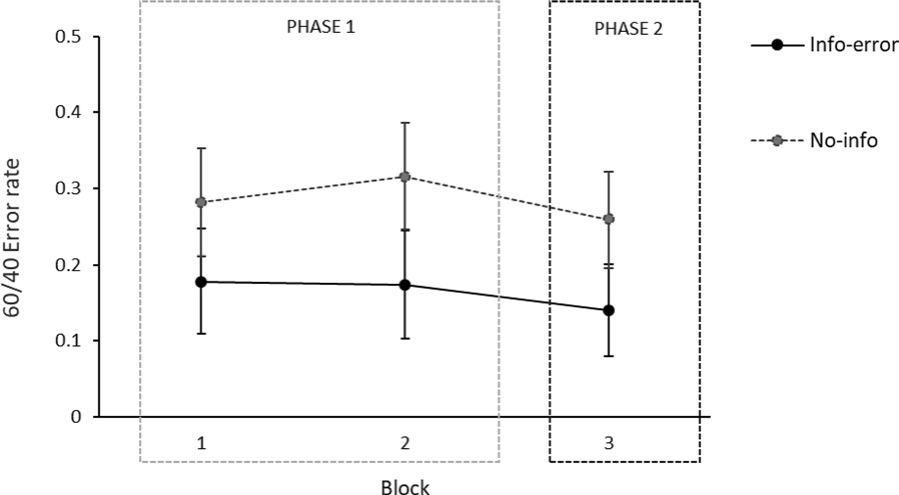
Participants’ mean error rates on each of the three blocks of 60/40 trials of the classification task for info-error and no-info groups in Experiment 2. Error bars depict the 95% confidence intervals for the means

Since previous analyses revealed no significant effects involving error, false positive or false negative, this factor was collapsed in subsequent analyses to further examine the effect of warning information. Figure 7 reports the mean number of 60/40 misclassification errors participants made in the AI-assisted (Blocks 1 and 2) and unassisted (Block 3) phases of the classification task as a function of the information participants received at the beginning of the experiment (informed about AI error or non-informed).

Overall, our results suggest that providing general information about potential AI errors prompted participants to be more cautious when accepting its recommendations, as reflected in fewer errors in the 60/40 cases for the info-error group compared with the no-information group (see Fig. 7). During Phase 1, participants who were informed about AI errors followed the AI’s recommendations to a lesser extent than those who were not informed, which mitigated bias replication in Phase 2.

In Experiment 2, the mean ratio of biased classifications of the ambiguous 50/50 samples in the info-error group was 0.59 (*SD* = 0.39) and in the no-info group was 0.66 (*SD* = 0.39). The differences between the groups were not statistically significant, *t*(196) =  − 1.09, *p* =.276. This suggests that the information about AI errors displayed at the beginning of the task did not introduce differences in the way participants classified the 50/50 samples in Phase 2. We found no evidence that the warning message had impact on the generalisation of the acquired bias.

A total of 93% of participants in the info-error group reported having detected errors in the AI results when they were asked about it at the end of the classification task, while this rate dropped to 81.6% for the no-info group. The differences between the groups were significant as confirmed by a chi-squared test χ^2^(1,198) = 5.80, *p* =.016. Participants who reported detecting errors were also asked to estimate the error rate they perceived in IA recommendations. Participants in the info-error group estimated that on average 24.4% (SD = 17) of recommendations made by the AI were erroneous, while the percentage estimated by the no-info group was on average 20.5% (SD = 14.6). The difference between the estimates of the two groups was not statistically significant, *t(*166) = 1.58, *p* =.117, *d* = 0.244. As we observed in previous experiments, in Experiment 2 those participants who tended to accept the biased AI advice more freely during the AI-assisted phase (Phase 1), making more errors in the classification of 60/40 samples, also tended to find the AI suggestions as more helpful, *r* =.43, *p* <.001, reported to have followed more the AI advice, *r* =.49, *p* <.001, and were more likely to trust AI applied to the health domain, *r* =.35, *p* <.001.

## Experiment 3: An Additional Control Condition

The influence of error type is an intriguing possibility that could help explain the unexpected results observed in Experiment 1 regarding the different instruction versions. However, our primary evidence for the differential impact of false positives versus false negatives on participants’ response tendencies is based on exploratory post hoc comparisons and descriptive observations of the results of Experiment 2. As these provided only limited evidence for the involvement of the type of error in shaping participants’ behaviour, further investigation was needed. Furthermore, Experiment 2 did not include a group that did not receive support from the AI system or any other type of assistance. Adding a control condition without interaction with the AI outputs would allow us to assess whether the observed effects are due to interaction with the system or whether pre-existing biases or participants’ response tendencies contribute to the findings.

To address these open questions, we conducted Experiment 3, a pre-registered replication of Experiment 2 (pre-registration: https://aspredicted.org/55yu76.pdf) with two specific aims. First, we wanted to test the robustness of the trends observed in the false-positive and false-negative conditions in the previous experiment. This replication allowed us to further investigate the effects associated with different error types. Second, we introduced an additional control condition to disentangle the specific impact of the warning message on potential AI errors in bias acquisition from other potential response tendencies that could impact the results. This was achieved by comparing groups that interacted with the AI, either with or without information about potential errors, with a group that neither interacted with the AI system nor received any other form of support during the classification task.

## Method

### Participants

The final sample consisted of 243 participants (mean age = 34.9 years, *SD* = 11.9; 53.1% female, 46.5% male, 0.5% non-binary) recruited via the Prolific platform. Participants received £1.30 for completing the experimental task, which took approximately 13 min on average. Initially, 254 participants completed the task. After applying the preregistered exclusion criteria (identical to those used in Experiments 1 and 2), we excluded 9 participants who failed to achieve at least five correct responses in the repetition of the practice phase and 2 participants who did not perform above chance level (i.e., fewer than 30 correct responses) in Phase 1.

A post hoc sensitivity analysis conducted using G*Power indicated that, with a sample size of 243 participants, the study had 80% power to detect an effect size as small as *f* = 0.18 in a repeated-measures ANOVA with a between-groups factor.

### Procedure

The procedure in Experiment 3 was identical to that of Experiment 2, with the exception of the inclusion of an additional control condition. Therefore, the main novelty of Experiment 3, relative to the two prior experiments, was the addition of a group that was informed about the potential errors of AI systems but did not receive AI support during the classification task.

After completing the practice phase, participants in Experiment 3 were assigned to one of three groups: info-error AI, no-info AI, and info-error unassisted, with the latter serving as the additional control. Within each group, participants were randomly assigned to one of two instruction versions (B1 or B2), such that they were randomly exposed to an AI system that systematically produced false negatives (B1) or false positives (B2). As in Experiment 2, the simulated AI in Experiment 3 made errors always in 60/40 samples.

**Fig. 8:**
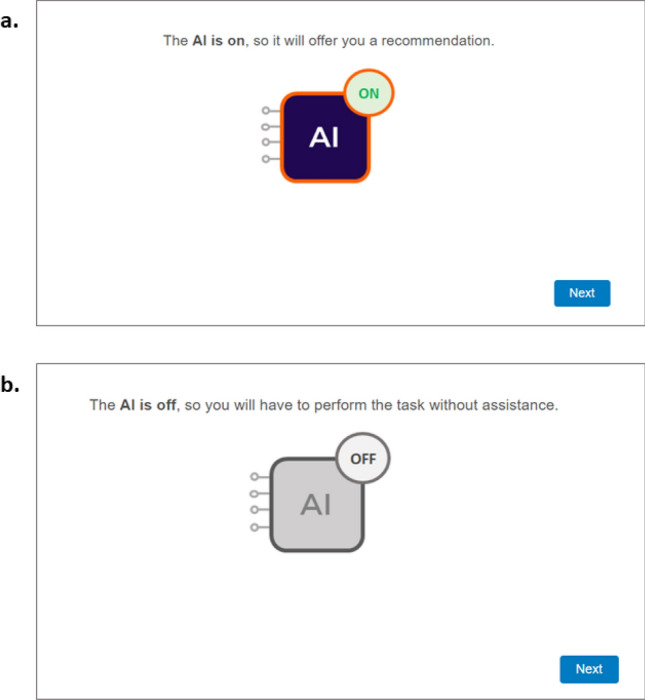
Screenshots showing the message indicating whether the AI system was on or off in each phase of the classification task in Experiment 2. **a** indicated that the AI was switched on for the AI-assisted groups in Phase 1, while **b** was displayed at the beginning of Phase 2 to show that participants in the AI-assisted groups would have to classify independently, as the AI had been switched off. Participants in the unassisted group received the same indication that the AI was off at the beginning of Phases 1 and 2

The instructions and the error warning message were identical to those used in Experiment 2. However, we added a clarification to specify when participants did and did not receive AI support. Before starting the classification task, participants were informed: “If the AI is on, it will offer you a recommendation that will appear at the top of the screen above the sample, as shown in the image. If the AI is off, you will have to perform the task without assistance”. In the AI-assisted groups, a brief message indicated that the AI was on during Phase 1 and off during Phase 2 (see Fig. 8). In the unassisted group, participants were informed that the AI was off at the beginning of both Phase 1 and Phase 2.

Regarding the post-experimental questions, Experiment 3 included the same items as Experiment 2, with a minor adaptation. Specifically, the first question asked, “Has the AI algorithm been connected and have you received recommendations at any point during the task?” This question assessed whether participants correctly understood whether an AI system was providing recommendations (in the AI-assisted groups) or not (in the unassisted group).

### Results and discussion

As preregistered for Experiment 3, we conducted a mixed ANOVA with group (info-error AI, no-info AI, info-error unassisted) and type of error (false negative, false positive) as between-subjects factors and block (Block 1,2 and 3) as a within-subjects factor on the mean error rate in 60/40 trials for each block of five trials. Blocks 1 and 2 consisted of five 60/40 trials from Phase 1, whereas Block 3 comprised the five 60/40 trials from Phase 2.

The analysis revealed a main effect of group, *F*(2, 237) = 20.076, *p* <.001, *η*^2^*ₚ* =.145, and a main effect of block, *F*(2, 474) = 15.937, *p* <.001, *η*^2^*ₚ* =.063, as well as a Group × Block interaction, *F*(4, 474) = 7.111, *p* <.001, *η*^2^*ₚ* =.057. No main effect of type of error was observed, *F*(1, 237) = 3.578, *p* =.060, *η*^2^*ₚ* =.015, nor were there significant interactions involving type of error with group, *F*(2, 237) = 0.482, *p* =.618, *η*^2^*ₚ* =.004, or with block, *F*(2, 474) = 0.065, *p* =.936, *η*^2^*ₚ* <.001. The three-way Group × Block × Type of Error interaction was also not significant, *F*(4, 474) = 0.341, *p* =.851, *η*^2^*ₚ* =.003.

Therefore, we did not observe a significant impact of the type of error (false positive versus false negative) on participants’ responses. Consequently, these results do not provide robust evidence for a trend towards a higher number of errors in the false-positive condition than in the false-negative condition, as suggested by the post hoc comparisons in Experiment 2. Since the type of error did not show a statistically significant effect in the previous analysis, we decided to collapse that variable. Therefore, we performed a 3 (Group) × 3 (Block) ANOVA to look in detail only at the variables with evidence of an effect. The ANOVA revealed a main effect of group, *F*(2,240) = 20.4, *p* <.001, *η*^*2*^_*p*_ =.145, a main effect of block, *F*(2,480) = 16.05, *p* <.001, *η*^*2*^_*p*_ =.063, and a Block × Group interaction, *F*(2,480) = 7.36, *p* <.001, *η*^*2*^_*p*_ =.058.

**Fig. 9:**
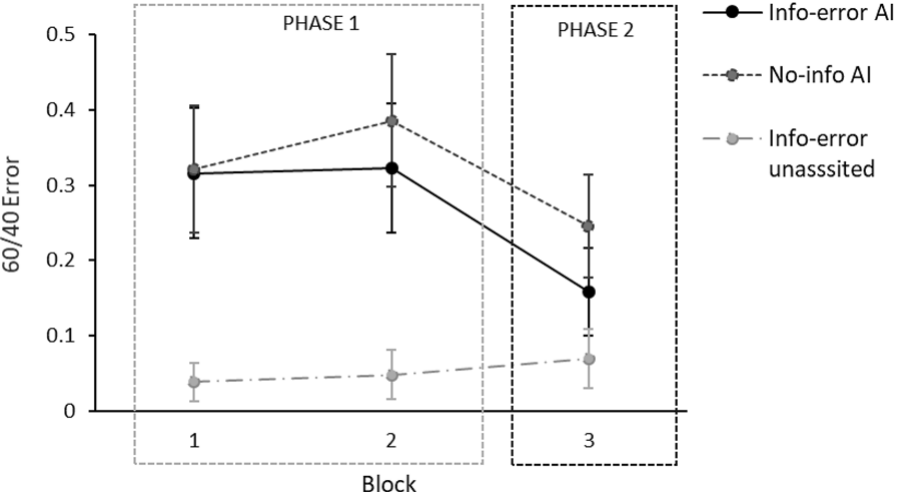
Participants’ mean error rates on each of the three blocks of 60/40 trials of the classification task for each group in Experiment 3. Error bars depict the 95% confidence intervals for the means

Figure 9 presents the mean error rates for 60/40 trials across blocks for each experimental group throughout the classification task. The figure shows a clear difference between the group without assistance and the two AI-assisted groups. In contrast, no clear difference in mean 60/40 errors is observed between the two AI-assisted groups (info-error AI and no-info AI). Participants who were warned about potential AI errors exhibited only a slightly lower error rate than those who were not warned, and this difference does not appear to be statistically significant. Additionally, the AI-assisted groups showed changes in error ratio across blocks, with an increase from Block 1 to Block 2, followed by a decrease from Block 2 to Block 3.

In Block 3, we did not observe a significant difference in mean error rates between the info-error AI and no-info AI groups (see Fig. 9). Notably, in Block 3, during which all groups performed the classification task without AI assistance, post hoc comparisons with Tukey correction showed no evidence of a significant difference between the info-error AI group and the info-error unassisted or control group, *t*(240) = 2.08, *p*_*tukey*_ =.485, in contrast to the pattern observed in Blocks 1 and 2. By contrast, post hoc analyses revealed a significant difference between the no-info AI group and the info-error unassisted group in Block 3, *t*(240) = 4.17, *p*_*tukey*_ =.001.

We interpret these results as indicating that information about potential AI errors mitigated the tendency to reproduce the model’s errors when participants who had previously received AI recommendations were required to make decisions independently. Specifically, participants in the info-error AI group did not differ significantly in their performance in Phase 2 (without assistance for all groups) from participants who had not interacted with the AI at any point during the task. In contrast, the difference in error rates between participants who received AI assistance without being warned about potential errors (no-info AI) and the control group remained significant in this final phase. Although the no-info AI group showed a reduction in error rate once the AI was disconnected and participants classified independently, they still made significantly more errors than the unassisted group. This pattern demonstrates the persistence of AI bias and replicates the effect observed in previous studies.

We also examined the effects of Group and Error Type on the participants’ tendency to classify ambiguous 50/50 samples in the same direction as the AI bias during Phase 2 using a 3 (Group) × 2 (Error Type) ANOVA. The analysis revealed a main effect of group, *F*(2, 237) = 11.87, *p* <.001, *η*^2^*ₚ* =.091, but no main effect of error type, *F*(1, 237) = 2.05, *p* =.153, *η*^2^*ₚ* =.009, and no Group × Type of Error interaction, *F*(2, 237) = 0.08, *p* =.919, *η*^2^*ₚ* =.001.

**Fig. 10:**
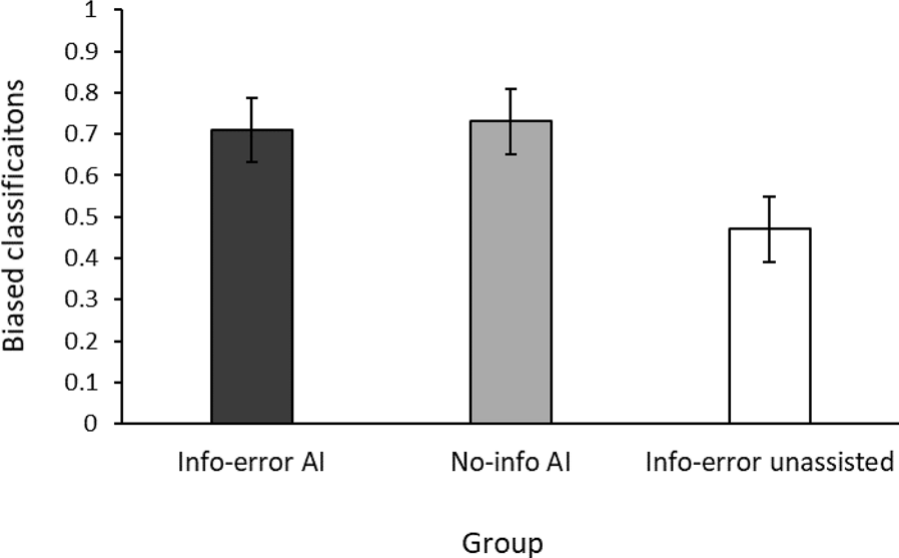
Participants’ mean biased classifications of 50/50 cases for each group in phase 2 of the Experiment 3

As shown in Fig. 10, participants who had previously interacted with the AI’s recommendations showed a stronger tendency to classify ambiguous 50/50 cases in the direction of the AI’s bias than participants who completed the task without any assistance.

As shown in Table 6, participants assisted by AI made more errors on samples with 60% dark-coloured cells and 40% light-coloured cells (cases in which the simulated system provided incorrect recommendations) compared with their responses to stimuli with different proportions.

**Table 6:** Mean error rates (and standard deviations) for all stimuli of different proportions of dark/light cells used in the classification task in experiment 3

Grupo	Phase 1						Phase 2			
	80/20	70/30	60/40	40/60	30/70	20/80	70/30	60/40	40/60	30/70
Info-error AI	0.00 (0.01)	0.00 (0.01)	0.32 (0.38)	0.05 (0.20)	0.01 (0.09)	0.00 (0.01)	0.03 (0.14)	0.15 (0.27)	0.08 (0.24)	0.04 (0.20)
No-info AI	0.00 (0.03)	0.01 (0.04)	0.35 (0.39)	0.03 (0.13)	0.00 (0.05)	0.00 (0.02)	0.04 (0.12)	0.24 (0.32)	0.06 (0.20)	0.02 (0.11)
Info-error unassisted	0.00 (0.04)	0.00 (0.04)	0.04 (0.12)	0.13 (0.28)	0.02 (0.12)	0.00 (0.01)	0.00 (0.05)	0.06 (0.18)	0.13 (0.30)	0.04 (0.18)

The misclassification rate per stimulus class is the total number of classification errors made on stimuli of a given class divided by the total number of such stimuli in each phase of the experiment

Participants in the unassisted condition made few or no errors across the different case types, as evidenced in Table 6. The negative impact of AI assistance on the classification of 60/40 cases is particularly evident when contrasted with the good performance observed in the unassisted condition. Among participants who classified without any support, the highest error rate was observed for the 40/60 samples. One possible explanation is that these cases were more difficult to discriminate perceptually. Specifically, despite consisting of 40% dark cells and 60% light cells, the darker cells may have appeared more salient, creating the impression of a higher proportion (Schoenlein et al., 2022). However, this potential explanation does not fully account for the effect observed, which was not present in prior studies using exactly the same procedure (Vicente & Matute, 2023; Vicente et al., 2025). Furthermore, to address potential perceptual influences, the two balanced versions of the instructions emphasised either a majority of dark cells (B1) or a majority of light cells (B2).

In Experiments 2 and 3, AI bias was systematically associated with the 60/40 cases, rather than the 40/60 cases as in Experiment 1. There was also no evidence of perceptual effects associated with the 60/40 samples, nor any evidence that participants’ responses were influenced by the type of AI error (false positive or false negative) or the version of the instructions (B1 or B2). Therefore, the results of Experiment 3 suggest that pre-existing response tendencies or biases in participants are unlikely to account for the effects related to the influence of the simulated AI recommendations and warning message.

Regarding the post-experimental questions, the first that participants had to answer was whether or not the AI had been switched on and offered them recommendations. As expected, most of those in the AI groups chose the “Yes” option, while most of those in the non-AI group chose “No”. However, we found some incorrect answers: 1.85% of people in the info-error AI group and 2.38% of the no-info AI group incorrectly answered that the AI had not connected, even though it had. Meanwhile, 5.12% of the control group answered affirmatively to the same question, despite not having received any recommendations from the system. Additionally, 84.85% of participants in the info-error AI group and 81.25% of participants in the no-info AI group claimed to detect errors in the AI recommendations. They estimated an average error percentage of 20% and 19.3%, respectively, which is very close to the actual percentage of 16.7%.

Participants who tended to follow the biased recommendations of the AI during Phase 1, making more errors in the classification of the 60/40 samples, also tended to consider these model recommendations more useful, *r* = 0.470, *p* <.001, reported following the AI’s advice more, *r* = 0.545, *p* < 0.001, tended to consider the AI more accurate, *r* = 0.374, *p* <.001, and were more likely to trust AI applied to the field of health, *r* = 0.421, *p* < 0.001.

The nonparametric Kruskal–Wallis test revealed differences between the groups in the trust placed in AI in the field of healthcare, *χ*^2^ (2) = 7.53, *p* =.023, with a lower mean estimate in the info-error unassisted group, *M* = 5.79, *SD* = 1.69, than in the info-error AI group, *M* = 6.24, *SD* = 1.95, and in the no-info AI group, *M* = 6.31, *SD* = 1.82.

To conclude, we summarise the main findings of Experiment 3, which replicated the error-mitigating effect of warning information on potential AI errors but did not provide clear evidence of the impact of the different error types (false positive and false negative) on participants’ response tendencies. However, the main novelty of Experiment 3 was the inclusion of an additional control group. Experiment 3 extends the results of Experiments 1 and 2 by demonstrating that warning information mitigates participants’ replication of AI bias when making decisions independently. Nevertheless, they are still influenced by erroneous recommendations when making decisions with this support, as evidenced by their higher error rate in the classification task compared to unassisted participants. Additionally, the behaviour of unassisted participants enabled us to rule out the influence of pre-existing response tendencies in the classification of 60/40 cases, thereby demonstrating the robustness of the results of Experiment 3.

## General discussion and conclusions

Based on the results obtained in our three experiments, we can conclude that messages warning users about errors or biases in AI recommendations could help to prevent the influence of erroneous AI recommendations on their decisions as well as the inheritance of AI biases. Providing precise information about AI reliability emphasising the rate of error, in Experiment 1, and a general warning about possible AI errors, in Experiment 2, helped participants to calibrate their reliance on the AI model. Informed participants exhibited fewer errors in the classification task and followed biased AI recommendations to a lesser extent than non-informed participants. This prevented them from learning the AI classification strategy and mimicking the same systematic error exhibited by the AI model. In Experiment 3, the general warning reduced participants’ tendency to replicate AI bias when making decisions independently. However, the effect of the message was limited during the AI-assisted phase of the classification, as it did not prevent participants from following erroneous AI suggestions.

Our research findings represent a contribution to the currently limited knowledge about strategies to promote optimal human-AI collaboration. Prior studies found that informing people about AI errors did not necessarily reduce their compliance with erroneous machine-generated recommendations (Kupfer et al., 2023; Parasuraman & Manzey, 2010; Vered et al., 2023). Vered et al. (2023) informed their participants of the limitations and potential sources of error of the AI. Despite being warned that the AI advice was not always correct and could be modified or contradicted, participants still followed the AI’s incorrect recommendations. However, Vered et al. (2023) found that participants who initially had a negative impression of AI were more reluctant to accept AI advice. Yet promoting negative attitudes towards AI is an intervention that has also failed to achieve consistent success in preventing people from accepting incorrect AI advice in different studies (Rezazade Mehrizi et al., 2023). We found that informing participants about potential AI errors at the beginning of the task effectively reduced their tendency to follow erroneous AI advice, at least in a simple health-themed classification task. Differences in the experimental settings, tasks and procedures could account for the inconsistencies between our study’s results and some of the previous ones. We cannot be certain about the critical factors that could explain why a strategy works well in one type of task or context but not in another. More research is necessary to discover these factors.

Similarly, in Experiment 1, reporting AI reliability information as a percentage of correct classifications (i.e., 80%) did not prevent participants from following the AI’s biased suggestions. It seems that participants considered the 80% accuracy level as good enough to rely on the system (Yu et al., 2019). In contrast, reporting the AI’s 20% error rate reduced the influence of biased recommendations on participants’ decisions. Highlighting the error encouraged people to monitor AI more carefully. Therefore, our results show that it is relevant to consider the effect of information framing, a robust effect in psychology that has been demonstrated in multiple contexts (Kahneman, 2003; Levin et al., 1998; Tversky & Kahneman, 1974), also when determining how to report information on an artificial intelligence tool (Kim & Song, 2020, 2023; Lacson et al., 2005).

In Experiment 2, participants who were alerted of the possible presence of errors in the AI recommendations by a general warning message made fewer misclassifications of 60/40 samples (those in which the AI systematically made an incorrect suggestion) compared to the uninformed group in the AI-assisted phase of the task. This intervention also prevented them from learning and replicating AI bias in their future independent decisions. However, in Experiment 3, we observed that the warning message had limited effect on preventing participants’ errors in Phase 1 of the classification task. Among participants with AI support, there were no significant differences in error rates between those who were warned and those who were not. Furthermore, both AI-assisted groups made significantly more classification errors than the unassisted control group. Therefore, while a simple warning message may offer some protection against reproducing and learning AI biases, more extensive or salient interventions may be necessary to ensure an effect.

Experiments 2 and 3 also explored the influence of the type of AI error, false positive or false negative, in an attempt to understand the differences observed in participants’ behaviour between instructions B1 and B2 in Experiment 1. Individuals tended to make more errors in the false positive condition, a tendency that seemed consistent in the two experiments, regardless of the specific false positive error association with the B1 or B2 version of the instructions. However, Experiment 2 and Experiment 3 did not found evidence for a robust impact of the type of AI error on participants’ behaviour.

Our initial hypothesis, which motivated us to explore the effect of the type of error, was that participants would generally prefer to classify the tissue sample as positive when in doubt, thereby following the erroneous AI recommendation in some cases. This response tendency could stem from the belief that the false positive could have less severe consequences than a false negative in the medical setting simulated in our experiments. Conversely, a false negative (i.e. failing to identify a patient with the disease) could entail more serious consequences. In this latter condition, participants would judge the AI recommendation more critically and made fewer errors.

One possible explanation for the absence of differences between AI error types in Experiments 2 and 3 is that participants may not have weighted false positives and false negatives differently. In real medical settings, physicians typically assign different significance to these two types of error because they have different consequences. However, our participants were non-experts performing a simulated medical task, and we did not explicitly assess their interpretation of these errors. Future research could address this issue by measuring participants’ interpretation directly (e.g. through explicit judgements) or by manipulating the task structure to give the two error types distinct meanings, costs or incentives.

In sum, although we found no evidence to suggest that the type of error made by AI affects participants’ willingness to follow its recommendations or the effectiveness of the warning message, we believe that this series of experiments generates an intriguing hypothesis that could produce interesting results in future studies. Some academics have suggested that the type of errors made by AI could be as important as its accuracy or error rate. This highlights the need to explore how different types of errors (e.g. false positives versus false negatives) influence users’ perception and acceptance of AI models (Kocielnik et al., 2019; Kozyreva et al., 2023; Molina & Sundar, 2022; Rice & McCarley, 2011).

One of the strengths of our study is that the design and procedure of the three experiments described in the manuscript are based on a methodology that was previously tested and refined in four experiments (see Vicente & Matute, 2023; Vicente et al., 2025). These studies specifically examined participants’ vulnerability to learning and replicating the systematic errors displayed by AI when making decisions independently. This effect was observed in several experiments with different control conditions. For example, this included a control group without exposure to AI results (within-group) or a counterbalanced order of phases with and without AI support among participants (between-group).

Experiment 3 in this article replicates the effect observed in previous experiments, providing strong evidence of the impact of the AI’s response pattern on participants’ subsequent independent decision-making. However, the main objective of the three experiments presented in this article was not to replicate previous results, but rather to investigate whether warning people about potential AI errors could prevent them from making the same systematic mistakes, thereby offering a preventive measure against the inherited bias effect observed in previous studies (Glickman & Sharot, 2024, 2025; Vicente & Matute, 2023). Therefore, our series of three experiments makes a novel contribution.

Regarding the experimental procedure, it should be noted that the diagnostic task used in our study was relatively easy. Vicente and Matute (2023) reported the error rates of humans alone in this type of task, which ranged between 0.00 and 0.06, indicating that participants were extremely accurate and could perform the task perfectly without assistance. This raises the question of whether it makes sense to use AI assistance for an easy task like this in real-world settings. However, many AI systems are being used in contexts where the technology does not yet outperform human decision-making. The reason is that AI is not always introduced to improve human decisions, but also to allow humans to make a lot more decisions in less time (e.g., Chen et al., 2024), to mitigate the effects of fatigue when sustained attention is required for long periods of time, and even to reduce employment costs by reducing the number of human workers necessary for a given task. Moreover, people may even follow AI advice against their own in easy tasks, in order to avoid conflict and potential errors for which they would be held accountable if they contradict the AI’s recommendations. Thus, it is important to investigate the quality of human-AI collaboration not only in cases where the human-AI team outperforms humans alone but also when humans could complete the task perfectly on their own (Vaccaro et al., 2024). This is because AI can sometimes reduce the quality of independent human work. In our experiments, the system’s biased recommendations still influenced participants’ performance in the easy task that we used. Importantly, however, the results show that this effect could be mitigated by warning users of potential errors beforehand. We believe that this strategy of reminding users of the fallibility of AI is important because may help them better decide when to use the system and when to rely on their own judgement.

Current regulations governing the use of artificial intelligence mandate that high-risk AI systems need to be designed and developed in a manner that enables the person to whom human oversight would be assigned, to understand how the AI system works, with its strengths and limitations, to correctly interpret AI output and even to remain aware of their human tendency to over-rely on AI (Article 14 of the Artificial Intelligence Act, Council of the European Union, 2024). That is, the AI system need to be easy to monitor and facilitate the human the decision to override or reverse AI outputs in any given situation. More knowledge is needed on what are the best approaches to promote adequate human oversight and not just to meet regulatory requirements. Simply having humans involved in the process will not be enough to ensure safety if their oversight is not meaningful. Unfounded assumptions about the ability of human users to effectively oversee AI can have a myriad of negative consequences, including reduced accountability of developers, companies, and government agencies for any harm caused by the technology (Green, 2022; Laux, 2023).

Machines are not infallible. AI can make errors and be biased. When humans interact with an AI they can adopt its biases (Vicente & Matute, 2023). It is important to note that bias in artificial intelligence should not be judged in moral terms, as it is a mathematical or statistical artefact. However, such bias in an AI model can lead to discrimination, unfair choices, or prejudice against a person or group (High-Level Expert Group on AI 2019), depending on how AI recommendations and predictions are used and the contexts in which they are applied. Machines are not sentient beings and cannot discern whether a decision is ethical or not (Véliz, 2021). For this reason, the human supervisor is trusted to discern whether a particular decision is ethical or fair. Increasing human control over AI seems to be the panacea for every potential risk associated with the use of this technology (Ryan, 2024). How can a human in the loop be the ultimate solution when humans can see their biases reinforced or acquire new biases when interacting with AI?

Based on our findings, we can suggest some recommendations on how to communicate information about artificial intelligence responsibly, so that users can have a clear idea of the risk of error or biases in the tool they are going to work with. When it comes to providing information about an AI’s accuracy, people may be better able to interpret this information if the model’s error rate is highlighted. Similarly, even simpler and more general information that emphasises the fallibility of AI in general would also reduce people’s tendency to accept biased recommendations from an AI. Both strategies have also proven to be effective in preventing people from acquiring biases through their interactions with machines. However, there is likely no one-size-fits-all strategy to protect humans from the risks posed by the use of AI-based tools, as evidenced by the disparity in research findings. Strategies should be adapted to the characteristics of the end user, the domain in which humans and machines are collaborating, and other aspects of the collaboration context (Araujo et al., 2020; Bogert et al., 2021; Fahnenstich et al., 2024; Hudson & Franklin, 2023; Naiseh et al., 2023; Rago et al., 2021).

Warning messages regarding AI performance should be precisely aligned with actual model accuracy to ensure transparent communication and prevent the formation of misleading perceptions. Such warnings can significantly influence users’ preferences for AI models. Gaba et al. (2024) found that when an explicit warning about bias was paired with an actual fair model, participants were less likely to trust it. Explicit bias warnings should be applied with caution, as informing users that a model may be biased can outweigh the influence of the model’s demonstrated performance (Gaba et al., 2024). Thus, warning messages intended to reduce over-reliance on AI systems may inadvertently foster excessive distrust, even in contexts where AI models outperform human decision-makers.

More research is needed to find out what factors determine that a particular type of information, or a particular way of presenting information to users, would be effective in mitigating the negative influence of AI erroneous recommendations on people’s decisions. There are no clear and simple answers, and it is necessary to analyse this complex issue from different perspectives. All actors involved in the problem, from developers and institutions to users, should work guided by ethical principles to get one step closer to fair AI.

## Data Availability

The datasets generated and analysed during the current study are openly available in the Open Science Framework at https://osf.io/h2gmz. Experiments were preregistered in Aspredicted. Links to the preregistration of Experiments 1, 2 and 3 have been shared in the Method section of each experiment, clarifying which aspects were preregistered.

## Footnotes

1 In this section, we only refer to the type of error (false positive, false negative) to better understand the data, because it is one of the main variables we tested in Experiment 2, along with whether or not to warn participants about AI errors. Therefore, the association of the variable type of error to B1 or B2 would be implicit and if necessary can be checked in Table 3 in the Method section of Experiment 2.
